# Mushroom and cereal β-D-glucan solid state NMR and FTIR datasets

**DOI:** 10.1016/j.dib.2021.107765

**Published:** 2021-12-24

**Authors:** Alexandra Kremmyda, William MacNaughtan, Dimitris Arapoglou, Christos Eliopoulos, Maria Metafa, Stephen E. Harding, Cleanthes Israilides

**Affiliations:** a Division of Food, Nutrition and Dietetics, School of Biosciences, University of Nottingham, Sutton Bonington Campus, Loughborough LE12 5RD, United Kingdom; b Institute of Technology of Agricultural Products, National Agricultural Research Foundation, 1, Sofokli Venizelou St., Lycovrissi 141 23, Greece; c National Centre for Molecular Hydrodynamics, Division of Food, Nutrition and Dietetics, School of Biosciences, University of Nottingham, Sutton Bonington Campus, Loughborough LE12 5RD, United Kingdom

**Keywords:** Mushroom, Oat, Wheat, Barley, Solid state CP/MAS NMR, FTIR, β-D-Glucan database

## Abstract

In the associated published article Kremmyda et al. (2021), the C1 region of the NMR spectra were examined in detail, as the C1 carbon was common to the crucial linkages in the glucan, and the most clearly separated region in the spectra. We provide the original measurement data here in topspin format, in order that different authors can examine the regions corresponding to the other carbons and in addition repeat our processing of the FID's using different parameters.

Mushroom, Cereal, Reference and other samples, such as salts and salt hydrates have been measured. A series of different experiments on individual samples is also given. All of the samples and experiments available in the database are summarised in the Table below.

We also provide an FTIR database in the form of an excel workbook with spectra in frequency/absorbence pairs.

## Specifications Table

**Table:** 

Subject	Food Science: Food Chemistry
Specific subject area	Solid state Cross polarisation/ Magic Angle Spinning Nuclear Magnetic Resonance and Fourier Transform Infra-Red databases of β-D-Glucan extracts from Mushrooms and Cereals.
Type of data	These are the data that were used for the specified publication. The NMR Topspin files consist of original Free Induction Decays and processed Spectra recorded on a range of natural products; mushrooms and cereals. Infra-red spectra in an excel workbook consisting of frequency/absorbence pairs are also provided.
How data were acquired	Solid State ^13^C CP/MAS NMR data were recorded on a 600 MHz Bruker with solid state MAS 2.5 and 4 mm rotor probes. Most spectra were recorded using a 4 mm probe however due to probe unavailability, several reference salt spectra were recorded using a 2.5 mm probe at the same frequency of 600 MHz and using the same method. Infra-red spectra recorded on a Bruker Tensor 27 with Spec-ac Golden Gate ATR accessory.
Data format	NMR Raw data and corresponding processed data are provided in the form of Topspin compatible files. The raw data can be completely reprocessed e.g. using different phasing and smoothing functions using the Bruker Topspin program or any other NMR processing programs which can read Bruker format files. FTIR data are provided in the form of excel compatible frequency/absorbence pairs all embedded in a single macro enabled workbook allowing comparison between 2 groups of spectra.
Parameters for data collection	See associated article [1] for details of the experimental conditions used for NMR data collection and for particular experiments. Briefly, this consisted of a parameter set containing all parameters required to run the spectrometer in the acquisition mode. The FID data set contained 4096 points with a dwell time of 10 usec. This gave an acquisition time of 0.04096 s and a sweep width of 50,000 Hz with a spectral resolution of 24.4 Hz. The number of scans was a variable according to available spectrometer time but had a minimum value of 2048. Exponential smoothing was normally 20 Hz but once again was variable according to spectral quality. FTIR data [2] were collected in a standard format on a Golden Gate attenuated total internal reflectance Spec-ac accessory, as interferograms at a resolution of 4 cm^−1^ and Fourier transformed with a zero filling factor of 2, using a smoothing algorithm.
Description of data collection	^13^C CP/MAS NMR spectra were recorded on a Bruker AVANCE III 600 NMR spectrometer with narrow bore magnet and a 4 mm triple resonance probe. Samples were spun at 10 or 12 kHz. Chemical shifts were referenced to the up-field line of adamantane (29.5 ppm) Approximately 50 mg of sample was contained in the 4 mm rotor. For FTIR data collection 3 independent samples were loaded on the golden gate ATR diamond window. Spectra were collected using a standard method [2]. Spectra were mean centred and normalised for comparison purposes.
Data source location	NMR data were recorded in the Biodiscovery Institute (BDI) University of Nottingham University Park Nottingham, NG7 2RD FTIR data recorded in the Food Nutrition and Dietetics department, School of Biosciences University of Nottingham Sutton Bonington Campus Nr Loughborough LE12 5RD, UK
	Mushroom samples were collected and identified in the EPIRUS region of Greece. Cereal samples were supplied by NAGREF GREECE Sample extraction and preparation were carried out in the Institute of Technology of Agricultural Products, National Agricultural Research Foundation, 1, Sofokli Venizelou St, Lycovrissi, 141 23, Greece
Data accessibility	Data are hosted in the Mendeley Data repository. Repository name: Mendeley Data Data identification number: (DOI): 10.17632/gbjc8dxw55.1, 10.17632/gdcdpfggj9.1, 10.17632/4fp4rnds5f.1 Direct URL to data: https://data.mendeley.com/datasets/gbjc8dxw55/1, https://data.mendeley.com/datasets/gdcdpfggj9/1, https://data.mendeley.com/datasets/4fp4rnds5f/1, Instructions for accessing these data: Data have no access controls and can be freely accessed. After having downloaded the complete folder of NMR files, this should be put into an existing Topspin data directory or put into a folder that is subsequently activated as a new data directory in the Topspin program (version 4.1.1). If this is carried out correctly, then when the directory is opened in Topspin, the identity of the file, the program used and all information - as well as all the raw and processed data will be available. Opening individual files to look at data can be problematical. There should be no problem downloading and using the FTIR excel workbook. However, this is a macro-enabled excel workbook. In order to compare groups of data or individual files, ensure that the macros are enabled.
Related research article	[1]

## Value of the Data

• This dataset is unusual in that it consists of a set of β-D-Glucans extracts from a wide collection of wild, naturally grown and formally identified mushroom and well-defined cereal samples. No isotopic enrichment is used.
• Any investigator who is interested in examining the types of β-D-Glucans that can be extracted from a range of identified natural mushrooms and cereals will potentially find this dataset useful. Some of the samples are contaminated with buffer salts but the carbohydrate signals are still well defined and clear to see.
• We have focused on a limited chemical shift range in the NMR spectra. Further analysis of the NMR dataset should reveal additional interesting properties and comparisons amongst glucans. We have dealt mainly with the properties of mushrooms. There is an extensive set of cereal recordings available in the dataset which remain to be analysed in detail; as well as variable contact time and non-quaternary suppression experiments in the NMR dataset which deserve more careful consideration.
• In addition, we supply an FTIR database for the dry mushroom and dry cereal samples with a macro enabled workbook, which allows mean centring and normalisation, and comparison between 2 groups of data.

## 1 Data Description

This Table 1 shows all the dry and hydrated sample experiments which are present in the database. CP: simple cross polarisation magic angle spinning. VC: variable contact time. NQS: non-quaternary suppression. Data is available in Topspin format. VC experiments, consisting of groups of spectra for each of the 16 contact times, are also available in Topspin format*.*

**Table 1:** The list of dry and hydrated samples from mushrooms and cereals and the reference samples used for the NMR and FTIR experiments.

	Dry	Dry experiments	Hydrated	Hydrated experiments
Cereals				

oat *Flega*	√	‏ CP VC	√	CP NQS VC
oat *Pallini*	√	CP	−	−
barley *Persephoni*	√	CP	√	CP NQS VC
barley *Thessaloniki*	√	CP	−	−
wheat *Gekora*	√	CP	√	CP
wheat *Acheloos*	√	CP VC	−	−

Mushrooms				

*Amanita caesarea*	√	CP VC	√	CP VC
*Hygrophorus marzuolus*	√	CP VC	√	CP VC
*Pleurotus ostreatus*	√	CP VC	√	CP
*Macrolepiota procera*	√	CP	√	CP VC
*Boletus aureus*	√	CP VC	√	CP VC
*Craterellus cornucopioides*	√	CP	√	CP VC
*Agaricus urinascens*	√	CP	−	−
*Cantharellus cibarius*	√	CP VC	√	CP
*Ganoderma lucidum*	√	CP	√	CP
*Morchella esculenta*	√	CP	√	CP VC

Other				

*Sodium acetate*	Sigma	CP	−	−
*Sodium acetate trihydrate*	Sigma	CP	−	−
*Citric acid*	Sigma	CP	−	−

*Notes:*

*Some of the spectra will need to be reprocessed due to integral and peak calculations being carried out. These have introduced some slight distorsions, which reduce the spectral intensity to zero at some points. A simple reapplication of the Fourier transform with a suitable smoothing factor will restore the original spectrum.*
*ppm assignment is carried out by adjusting the field to place the adamantane line at the correct value for a group of spectra. Other parameters, such as O1, are then maintained constant for that group. However for some of the the earliest recorded spectra ppm values were set relative to adamantane by adjustment of parameters. These scales may need adjustment. Similarly some of the VC data were used for spectral fitting and relaxation measurements rather than spectral presentation, and ppm values will similarly need adjustment if the presentation of spectra is required.*
*Several repeat measurements are availble for some samples indicated by the presence of b in the filename. The format for the Filename is as follows : Title1_title2_ref_b_hyd_CP_date (mushroom or cereal name_whether a reference sample or not_any repeats carried out_whether dry or hydrated_type of experiment_original date recorded)*
*Pleuroti Ostreatus, Citrinopileatus and L. Edodes were not part of the original series. However the Pleurotus Ostreatus, which was part of the study, is given the additional description « wild »*
*For the sample wheat Acheloos, spectra for several specified extraction pH and temperature conditions, specified in the filename were measured.*
*Reference materials inculding salts and reference oat spectra can be found in the cereal folder.*

### 1.1 Non-Quaternary suppression pulse sequences

Proteins and other compounds which can be found associated with β-D-glucans contain aromatic carbons which appear in the range 120–150 ppm.

Lignin is a structural polysaccharide and is associated with the sometimes, slightly woody stems of mushrooms. Many of the carbons in the aromatic groups of lignin, for example, contain no directly attached protons which means that the use of pulse sequences which suppress carbons with directly attached protons will lead to an enhancement of these often-weak signals. These pulse sequences suppress carbons with attached protons by essentially allowing the protons to de-phase the carbon signal during a delay period when no other irradiation is applied. Refinements of this basic simple method can lead to a significant enhancement of the quaternary carbons relative to other signals present in the spectrum.

## 2 Experimental Design, Materials and Methods

The database consists of simple 1-Dimensional spectra of β-D-Glucans from natural products and control samples.

### 2.1 Experimental design

One of the purposes of the work was to examine the spectra and look for features in the spectra which correlated with the beta glucan content as measured by independent enzymatic methods. In this sense, the response variables could be said to be the intensities of features in the spectra; for instance the intensity at 104 ppm, which is reported in much of the Scientific literature to be the predominant frequency of the C1in a β-D-glucan. We have concentrated on features in the C1 region and we now make available the database in order that other workers can examine features in other regions. There were however other aims of the experiments, such as examining the effect of hydration on the spectra in more detail than has hitherto been carried out.

With regard to the number of measurements, these were limited firstly, in some cases, by the amount of sample available, in particular for *Agaricus urinascens*, where sufficient sample was only available for one independent dry run, and secondly by spectrometer availability, which severely limited the number of repeats. However, if the database is examined there are examples of repeat measurements for *Boletus aureus, Craterellus cornucopioides, Macrolepiota procera* and *Amanita caesarea*. Generally the agreement between repeat spectra was good, however the contaminating peaks could show variation although not usually in the ppm shift, after appropriate adjustment had been made allowing for spectrometer drift.

### 2.2 Materials and methods

The materials used in this work were extracts from identified mushrooms and cereals. One extracted powdered sample was supplied for each mushroom and cereal . The extraction method used for the Cereals can be found in the original article [1] together with literature references.

The extraction of cereal β-D-glucans is not routine with a wide range of extraction methods present in the literature. The study of the physicochemical properties of isolated β-glucan fractions requires extraction procedures which optimize yield, purity and retain the integrity of the β-glucan molecule. For this reason, a hot water extraction procedure was used.  The cereal seeds were ground and sieved to a powder and a suspension made using NaOH (1 M). This was followed by stirring for 90 min at 45 °C. The suspension was centrifuged, and the supernatant adjusted to pH 3.4 using citric acid, followed by precipitation using ETOH, and placed in a refrigerator for 24 h.

The extraction method used for the mushrooms was according to the following method:

β-D-glucans were isolated using a slightly modified method originally used for *Pleurotus ostreatus* mushrooms, according to references in the original article [1]. The fruiting body of the mushroom was frozen at −20 °C, cut into small pieces, freeze dried at −75 °C for 24 h, lyophilized until a constant weight was reached and powdered. The powdered mushroom was then defatted with ethanol using a Soxhlet extractor for 8 h and the resultant residue immersed in 0.9% NaCl solution at 70 °C for 24 h. This was then centrifuged at 5700 rpm for 10 min to remove the water-soluble polysaccharide and the residue extracted with 1 M NaOH at 40 °C for 8 h. The supernatant was then neutralized by 1 M CH_3_COOH and the precipitate (β-d-glucan) was collected and washed with distilled water several times to achieve bleaching. β-D-glucan at this stage is referred to as β-D-glucan paste.

### 2.3 NMR methods

The rotors were filled with the powdered sample using a purpose designed funnel, and the sample packed tightly using a packing tool of close tolerance fit. Additional grinding to reduce particle size was unneccesary and there were very few cases of unsuccessful spinning due to rotor imbalance. The adamantane sample and reference salt samples were prepared in the same way. An automated Bruker system adjusted bearing and drive pressures, the compressed drive air being fed from a dried system, to acheive optimum spinning in a minimum of time. Automated sequences were occasionally used if a series of measurements were to be made, for example succesive single CP, Variable Contact and NQS experiments.

### 2.4 FTIR methods

Powdered samples in triplicate were placed on the diamond window of a single reflection Specac ATR system. Further grinding of sample was not required. Samples were compressed on the diamond window of the ATR using a screw driven anvil, by a torque wrench in order to ensure that all samples were compressed to approximately the same pressure and made good contact with the window. Data files were converted into excel compatible frequency/absorbence pairs for further processing.

## Ethics Statement

The authors declare that this submission follows the ethical requirements for publication in Data in Brief.

## CRediT authorship contribution statement

**Alexandra Kremmyda:** Writing – original draft, Methodology, Formal analysis, Supervision, Project administration. **William MacNaughtan:** Writing – original draft, Methodology, Formal analysis, Supervision, Project administration. **Dimitris Arapoglou:** Conceptualization, Writing – original draft, Writing – review & editing, Project administration, Methodology. **Christos Eliopoulos:** Conceptualization, Writing – original draft, Writing – review & editing, Project administration, Methodology. **Maria Metafa:** Software, Writing – review & editing. **Stephen E. Harding:** Software, Writing – review & editing. **Cleanthes Israilides:** Conceptualization, Visualization, Supervision, Project administration, Funding acquisition, Writing – review & editing.

## Declaration of Competing Interest

The authors declare that they have no known competing financial interests or personal relationships which have or could be perceived to have influenced the work on this article.
